# The Clinical Application and Progress of Mirogabalin on Neuropathic Pain as a Novel Selective Gabapentinoids

**DOI:** 10.1155/2023/4893436

**Published:** 2023-04-28

**Authors:** Hui Tang, Jing Lu, Yazhuo Duan, Dejun Li

**Affiliations:** ^1^Shandong Provincial Hospital Affiliated to Shandong First Medical University, Jinan 250000, China; ^2^Stem Cell Clinical Institute, Shandong Academy of Clinical Medicine, Jinan 250021, China; ^3^Gaotang County People's Hospital, China; ^4^Department of Intensive Care Unit, Baoshan People's Hospital, Yunnan 678000, China

## Abstract

**Background:**

Neuropathic pain is a complex sort of pain that is detrimental to individuals' health, both physically and mentally, but merely a small portion of them could witness pain alleviation. Mirogabalin, by distinctive binding characteristics of voltage-gated calcium channels, has won approval from the Japanese authority as a third member of gabapentinoids in Japan. Our review was aimed at encompassing the bulk of clinical research on mirogabalin, which included clinical trials, special considerations, coadministration studies, case reports, and cost-effectiveness studies.

**Methods:**

A review was carried out on a series of platforms, such as PubMed, MEDLINE, and Scopus, up to December 2021 using the keywords as follows: “mirogabalin OR mirogabalin besylate OR Tarlige OR DS-5565” AND “neuropathic pain OR Neuropathy.”

**Results:**

Mirogabalin demonstrated analgesic activity and manageable adverse reactions and provides a new alternative for individuals with PHN or DPNP in 3 phase II and 4 III trials. Mirogabalin alleviated pain markedly in comparison with placebo. Administration of mirogabalin on a long-term basis is a flexible dosage regimen for patients with PHN. It is noteworthy that mirogabalin should be administrated cautiously when combined with probenecid and cimetidine on account of a slight increase in pharmacodynamics effects of mirogabalin.

**Conclusion:**

The development of mirogabalin allows further optimization of individual treatment strategies so as to provide more therapeutic choices in this medical domain.

## 1. Introduction

Neuropathic pain (NeP) is a complex sort of pain due to various underlying causes, such as a lesion or disease in the somatosensory nervous pathways, impairing patients' mental health, leading to sleep disorder, and affecting the quality of life [1, 2]. According to the newly published International Classification of Diseases 11th Edition, neuropathic pain is further divided into peripheral and central types [3]. The disease has an incidence rate of roughly 7-8%, accounting for nearly a quarter of the population with chronic pain [4]. Merely 50% of individuals with NeP could see partial (some 30-50%) pain alleviation, whereas the majority complain of unsatisfactory pain regulation [5, 6]. Therefore, novel drugs with remarkable analgesic effect and meanwhile minimal adverse reactions are needed as an alternative for NeP therapy [7]. Currently, gabapentinoids, tricyclic antidepressants, and serotonin norepinephrine reuptake inhibitors are employed as first-line agents for NeP [8]. Gabapentinoids (primarily gabapentin and pregabalin) can produce an analgesic effect by weakening dorsal horn sensitivity through binding to the *α*2*δ* ligand of voltage-gated calcium channels (VGCCs) [9], which are strongly associated with neuropathic pain relief mechanism [10, 11]. Although gabapentinoid therapies are effective in NeP treatment, the common adverse effects of somnolence, encephalalgia, and dizziness limit the scope of their clinical application [12, 13].

**Figure 1:**
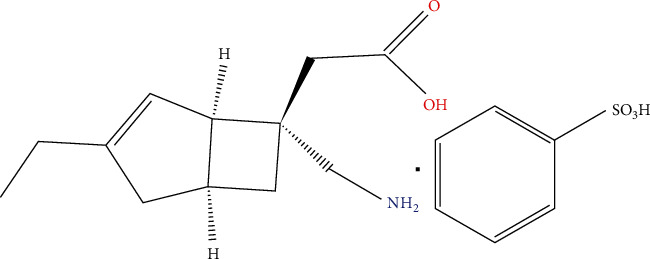
Chemical structure of mirogabalin.

Mirogabalin (Figure 1 and Table 1), as a third member of gabapentinoids, obtained approval in Japan in 2019, following completed phase II and III clinical trials on DPNP or PHN patients [14]. Subsequently, Korea, Taiwan, and China approved the application of mirogabalin as a cure for peripheral neuropathic pain and PHN and DPNP in the following year [15]. Patents were issued for mirogabalin by the European authority in September 2013 and by the American authority in May 2011 [16]. The *α*2*δ*-1 and *α*2*δ*-2 subunits of VGCCs have been identified as a target for therapies of gabapentinoid [17]. The *α*2*δ*-1 subunit of VGCCs is an enabling factor for analgesic effects of *α*2*δ* ligands [18, 19]. The contribution of the *α*2*δ*-2 to the central nervous system adverse reaction of *α*2*δ* ligands still needs to be elucidated [20]. The binding capability of mirogabalin for the *α*2*δ*-1 and *α*2*δ*-2 subunits was comparable to that of pregabalin. It nevertheless demonstrated a slower dissociation rate for the *α*2*δ*-1 than *α*2*δ*-2 subunits, notably for the *α*2*δ*-1 compared with pregabalin [16, 21]. Because of its distinctive binding features to *α*2*δ*-1 and *α*2*δ*-2, mirogabalin has superior analgesic efficacy and lower central nervous system- (CNS-) related adverse effects than pregabalin [22]. Therefore, the present study is aimed at critically covering the current clinical research on mirogabalin in treating NeP. To obtain a more comprehensive profile, we reviewed the extant literature in a systematic manner to summarize the advantages and disadvantages of mirogabalin when employed as a medication for NeP of different phases. Furthermore, the study discusses the strategy for combination therapy and other issues concerning mirogabalin application, such as case report and drug interaction along with special considerations.

## 2. Methods

**Figure 2:**
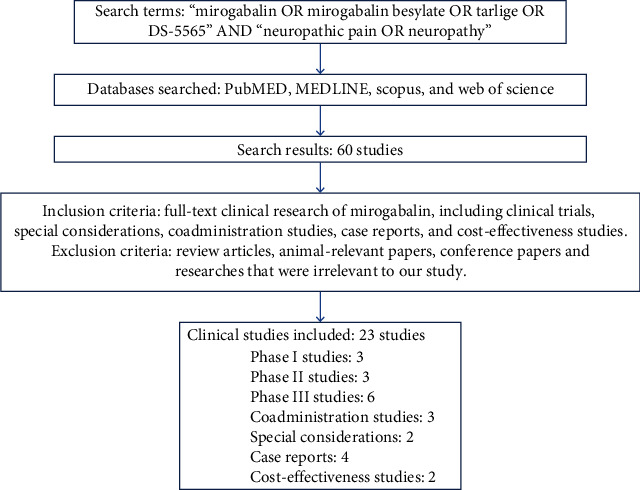
Search methodology.

A review was carried out to cover the existing clinical research on mirogabalin in the therapy of NeP, including pharmacokinetics, drug-drug interaction, case reports, and special considerations. PubMed, MEDLINE, Scopus, and Web of Science were searched through December 2021 using keywords as follows: “mirogabalin OR mirogabalin besylate OR Tarlige OR DS-5565” AND “neuropathic pain OR Neuropathy.” Articles were included if they were (i) all original phase I-III trials or (ii) case reports. Articles were excluded if they were (i) nonhuman studies, (ii) reviews, (iii) correspondence with editors, (iv) prospective or retrospective studies, and (v) beyond the scope of publication time or language.  Figure 2 provides detailed information regarding the search methodology adopted in the study.

## 3. Results

Altogether, three scores of articles were sorted out using the abovementioned search methodology. In accordance with the inclusion and exclusion criteria, 22 articles were selected for this review. Specifically, they consisted of 8 phase I studies including 3 drug-drug interactions and 2 special considerations studies, 3 phase II studies, 6 phase III studies, 4 case reports, and 2 cost-effectiveness studies.

### 3.1. Phase I Studies

#### 3.1.1. Pharmacokinetics (PK)

One phase I study assessed PK parameters and food effect of mirogabalin, and a cohort of 48 participants was enrolled and provided with six doses of 3, 5, 10, 30, 50, and 75 mg [23, 24]. Mirogabalin was rapidly absorbed and discharged, with *T*_max_ of 1.0 h and *T*_1/2_ values ranging from 2.96 to 4.94 h. Application of mirogabalin seemed to show a linear increment after single or multiple doses. Steady state was reached by day 3 for 5-20 mg, twice daily (BID) groups. The apparent total body clearance ranged between 16.50 and 18.24 L/h. In these subjects, 61%-72% of the dose remained unchanged before excretion in the urine, and renal clearance (CL_cr_) was 10.4-12.4 L/h. Multiple doses resulted in no considerable level of accumulation during the 14-day trial period. In the high-dose (50 and 75 mg) cohorts, it demonstrated graver impairment in PK assessments and increased occurrence rates of treatment-emergent adverse event (TEAE) than in well-tolerated dose (3-30 mg) cohorts. Somnolence as a TEAE was 66.7% and 50% in 50 mg and 75 mg cohorts, respectively. Mirogabalin can be taken without food restrictions, except the maximum plasma concentration (*C*_max_) value reducing by 18% and *T*_max_ delaying by 0.5 h under fed conditions.

Another study was a repeated dose study (10 and 15 mg BID) for 7 days and a single dose study (20 mg) in different countries [25]. The PK parameters of 20 mg/day mirogabalin were similar among Korean, Chinese, and white subject groups. *T*_max_ was almost 1 h or less, with *T*_1/2_ of 2-3 h in the single-dose study. Still, exposure seemed to increase in proportion to mirogabalin dose in repeated-dose groups. On Day 7, *T*_1/2_ was 2.4 and 2.8 h in 10 and 15 mg BID groups, respectively, and mean CL_cr_ was similar across dose levels, with 179.6 and 175.4 mL/min, respectively. No accumulation of mirogabalin in repeated dose study was detected. In the 53 subjects, somnolence (*n* = 17/53, 32%), encephalalgia (*n* = 11/53, 21%), and dizziness were frequently reported under the mirogabalin 20 mg dosage regimen.

Following the administration of mirogabalin labeled with ^14^C under a single-dose regimen (30 mg) in healthy male adults, ^14^C-mirogabalin was distributed into red blood cells, with a ratio of whole blood concentration to plasma concentration of 0.85 to 0.87 in human (*in vitro*) [26]. Nearly 96.8% of the radioactivity was recovered in the urine, and mostly 76.4% of the radioactivity in the urine was recovered as unchanged mirogabalin. The metabolite of mirogabalin found in urine, other than the unchanged mirogabalin, was the lactam form of mirogabalin and accounted for 0.6% of the dose.

#### 3.1.2. Special Considerations

A study in Japan investigated the drug security and pharmacokinetic changes of a single dose (5 mg) in 30 individuals with renal injury of different degrees. With CrCl^27^ as the parameter, renal function was categorized into five classes, namely, normal and impairment (mild, moderate, or severe), along with end-stage renal disease (ESRD). AUC_0-last_ of mirogabalin increased by 90% (geometric least squares means (LSM) (95% CI) and 1.90 (1.32-2.74)), 264% (3.64 (2.63, 5.23)), and 425% (5.25 (3.65, 7.55)), respectively, for patients with moderate, severe, and ESRD impairment, respectively, as compared to normal controls. In severe impairment and ESRD groups, the levels of the maximum plasma concentration (*C*_max_) values of mirogabalin were significantly higher (50% and 30%, respectively) than the normal group on the basis of geometric least squares (LSM) ratio. Total CL/F of mirogabalin was reduced by 25% (0.75 (0.54, 1.05)), 54% (0.53 (0.38, 0.74)), and 76% (0.28 (0.20, 0.39)) in subjects with renal damage, whether mild, moderate, or severe, in comparison with normal controls. In patients with end-stage renal disease requiring hemodialysis, 15.3% of dosed mirogabalin was removed from blood during a 4-hour hemodialysis. A clinical trial of mirogabalin in 32 individuals with mild or moderate hepatic impairment demonstrated that after a single dose of 15 mg of mirogabalin [27], the subjects showed different levels (105% and 84%, respectively) of *C*_max_ value of mirogabalin and different levels (89% and 108%, respectively) of AUC_inf_ than those in the contrast group.

#### 3.1.3. Drug-Drug Interactions (DDIs)

In total, there have been three published studies on drug-drug interactions with mirogabalin (Table 2). One phase I crossover study examined 30 healthy male grown-up volunteers and concluded that the PK of mirogabalin were not affected when mirogabalin (a single dose of 15 mg) was utilized alone or combined with metformin (850 mg). This demonstrated that mirogabalin in combination with metformin had no impact on the PK of mirogabalin [28]. *T*_max_ of mirogabalin was 2 hours when administered alone or combined with metformin. Geometric LSM ratios of *C*_max_, AUC_last_, and AUC_0-inf_ in the codrug group were 0.94 (codrug/single use, 90% CI (0.87, 1.02)), 0.99 (0.95, 1.04), and 1.00 (0.96, 1.04), respectively, in contrast with the single-drug group. Likewise, the combined drug administration exerted no considerable effect on the PK of metformin. A total of three subjects reported TEAE following coadministration, including dyspepsia, encephalalgia, and increased hepatic enzymes (AST and ALT were 2.7 and 2.5 × ULN). On the whole, the two drugs had good tolerability in healthy controls, indicating no obvious sign of drug-drug interactions.

Another clinical study investigated the impact of mirogabalin interaction in the presence of lorazepam, tramadol, zolpidem, or ethanol [29]. The statistical changes in *C*_max_ of mirogabalin were observed during coadministration with tramadol (geometric LSM ratios (90% CI), 0.72 (0.67, 0.76)), zolpidem (0.89 (0.82, 0.96)), and ethanol (1.20 (1.12, 1.28)), compared with mirogabalin. Exposure to interacting drugs was similar when taken alone or in combination with mirogabalin. In security evaluation, combined administration of mirogabalin with lorazepam or ethanol magnified the impact of body sway and digit symbol substitution test (DSST) assays, with reduced DSST scores compared with separate administration (*P* < 0.05). Combined administration of mirogabalin with lorazepam or with zolpidem increased the occurrence of somnolence, twice greater than separate administration of lorazepam (22.2%) and four times greater than separate administration of zolpidem (10.0%). Similarly, mirogabalin in combination with tramadol or ethanol raised the incidence rate of nausea and encephalalgia, respectively.

In a third phase I crossover study, 30 healthy adult volunteers were evaluated for the effect of probenecid 500 mg Q6H (metabolic clearance inhibitor) and cimetidine 400 mg Q6H (only renal clearance inhibitor) on mirogabalin (15 mg) exposure [30]. AUC_0-last_ and *C*_max_ of mirogabalin increased by 76% (geometric LSM ratios (90% CI), 1.76 (1.72, 1.80)) and 29% (1.29 (1.22, 1.36)), respectively, when combined with probenecid, and increased by 43% (1.43 (1.40, 1.47)) and 17% (1.17 (1.11, 1.24)), respectively, when combined with cimetidine. The mean standard deviation of CL_cr_ and CL/F was substantially lower for the combined regimen (probenecid or cimetidine) than for mirogabalin alone. For security evaluation, the 15 mg dose of mirogabalin was well tolerated, without serious adverse effects or adverse reactions resulting in drug discontinuation for mirogabalin or combined with probenecid or cimetidine.

### 3.2. Phase II Studies

In a double-blind, placebo- and active-controlled comparator phase II study (NCT01496365) of 452 patients with DPNP, subjects were randomly assigned (2 : 1 : 1 : 1 : 1 : 1:1) to placebo, mirogabalin 5 mg/day once a day (QD), mirogabalin 10 mg QD, mirogabalin 15 mg QD, mirogabalin 10 mg BID, mirogabalin 15 mg, BID, or pregabalin 150 mg BID [31]. Up to the fifth trial week, remarkable changes could be detected in average daily pain score in LSM contrasted with placebo at dose levels of 15, 20, and 30 mg/day (*P* < 0.05). The stated changes started from week 1 and sustained well into week 5 (*P* < 0.05). The commonest clinical adverse reactions of mirogabalin were dizziness (7.6%) and somnolence (5.1%), but the occurrence rate was lower than with pregabalin, whose adverse effects were reported to include somnolence, balance disorder, fatigue, and peripheral edema, with an occurrence rate of 8.0%, 4.0%, 4.0%, and 4.0%, respectively.

Focusing on the same objectives [31], a study evaluated self-reported pain and sleep disorder in DPNP [32]. In the mirogabalin 15, 20, and 30 mg/day groups, remarkable decreases in average daily sleep interference score were detected as compared to placebo (*P* < 0.05). The subjects were followed up for five weeks, and at week 5, the results suggested that the mirogabalin groups of different doses (a daily dose of 5, 10, 15, 20, and 30 mg, respectively) reported a markedly higher PGIC status (49.1%, 54.5%, 48%, 48.1%, and 50%, respectively) than the placebo group (31.1%). The differences were of statistical significance (*P* < 0.05).

In another phase II double-blind, randomized, and placebo-controlled study (NCT01504412), 450 participants with DPNP were randomly allocated to 5, 10, and 15 mg BID mirogabalin, 150 mg BID pregabalin, and placebo groups [33]. The primary endpoint improved in baseline changes in ADPS at week 7 in each mirogabalin group in comparison to placebo and pregabalin groups, though showing no statistical difference. The placebo-adjusted LSM difference in short-form McGill Pain Questionnaire (SF-MPQ) sensory, visual analog scale (VAS), and ADSIS were statistically significant in the 15 mg BID group (-1.9, 95% CI (-3.6, -0.2), -7.4, 95% CI (-13.0, -1.8), and -0.9, 95% CI (-1.3, -0.4)), respectively. The prevalent TEAEs associated with mirogabalin included somnolence (14.7%), dizziness (11.0%), and nasopharyngitis (8.4%). The occurrence of somnolence and dizziness increased with the increase of mirogabalin dose.

### 3.3. Phase III Studies

#### 3.3.1. DPNP

Baba et al. published a research based on a double-blind, placebo-controlled phase III study (NCT02318706) [34]. They followed up 834 patients with DPNP for 14 weeks under a dosage regimen of 15, 20, or 30 mg/day mirogabalin or placebo. At the end of the trial period, the LSM change from baseline in ADPS was -1.31, -1.34, -1.47, and -1.81 for the placebo and mirogabalin groups, respectively, which was statistically significant for mirogabalin 30 mg/day versus placebo (*P* = 0.0027). In addition, the reduction in VAS and in ADSIS was of statistical importance for mirogabalin 30 mg/day versus placebo (*P* = 0.0018 and *P* = 0.0001), respectively. As compared to placebo groups, markedly, more patients under the dosage regimen of 30 mg/day mirogabalin reported “minimal improvement” of PGIC (score ≤ 3, 70.3% vs. 58.8%, *P* = 0.0129) or “significant improvement” (score ≤ 2, 40.0% vs. 26.1%, *P* = 0.0016). The frequencies of adverse reactions were 42.4% (70/165) in the 20 mg/day group and 62.4% (103/165) in the 30 mg/day group. The observed adverse effects were nasopharyngitis, somnolence, dizziness, peripheral edema, and weight change. Serious dizziness and edema occurred in two subjects, and a severe TEAE of increased alanine transferase and hepatic enzyme occurred in one subject, and all these adverse effects vanished without being treated.

Another study focused on the security and efficacy of mirogabalin on a flexible dosage basis [35], and 214 DPNP patients were selected for the extension study after completing a double-blind study (NCT02318706) and meeting eligibility criteria. All SF-MPQ scales, including sensory score, affective score, total score, VAS, and present pain intensity, generally showed a declining tendency from the start to the final trial week. A total of 59 (27.6%) out of 214 DPNP patients underwent adverse reactions, and the commonest TEAEs include mild or moderate somnolence (7.9%), dizziness (6.1%), and peripheral edema (4.7%), with mild or moderate.

#### 3.3.2. *PHN*A

A placebo-controlled phase III study also examined the efficacy and security of mirogabalin [36] (NCT02318719). The participants were randomly assigned to groups of placebo or mirogabalin 15, 20, or 30 mg/day and followed up for 14 weeks. In the last trial week, the differences in ADPS for the mirogabalin groups were of statistical importance compared with placebo. The placebo-adjusted LSM difference was 20.41 (95% CI: (20.74, 20.07), *P* = 0.0170), 20.47 (20.81, 20.14) (*P* = 0.0058), and 20.77 (21.10, 20.44) (*P* < 0.0001) for mirogabalin 15, 20, and 30 mg/day groups, respectively. Furthermore, the LSM changes from baseline to week 14 in the SF-MPQ and the ADSIS were considerably greater in all study groups compared with placebo (*P* < 0.05). Patients in mirogabalin 15 mg/day group had significantly “improved or better (score ≤2)” PGIC at week 14 than those in the placebo group (36.2% vs. 26.4%, *P* = 0.0318). A considerable number of patients who received mirogabalin 20 and 30 mg/day reported a minimal improvement in PGIC score. All of these research results were similar to the previous study in 2017 [32]. The frequencies of adverse reactions were 35.3% (54/153) in the 20 mg/day group and 44.5% (69/155) in the 30 mg/day group. With the increase of the daily dose, the TEAEs increased. In the 15 mg/day group, pneumonia, rib fracture, and femur fracture were reported; in the 20 mg/day group, erectile dysfunction, fracture, and upper limb fracture were reported; in the 30 mg/day group, higher blood creatine phosphokinase, memory impairment, cerumen impaction, and electrocardiogram change were reported.

Another extension study founded on phase III study [36] investigated the subjects for 52 weeks and focused on the long-term security and efficacy of mirogabalin on a flexible dosage basis. A cohort of 239 participants went through the double-blind study (NCT02318719) and entered the extension study of 52 weeks. The whole trial was comprised of a titration period of four weeks, a dosage adjustment period of 48 weeks, and a follow-up period of one week. All SF-MPQ scales diminished from the start to the end. The reported adverse reactions included somnolence (13.5%), dizziness (10.1%), weight change (7.2%), edema (4.2%), and peripheral edema (2.5%). An abnormal 12-lead electrocardiogram was observed in 2 patients with great clinical significance: one case of atrial flutter and one case of acute myocardial infarction.

#### 3.3.3. DPNP or PHN with Renal Impairment

A phase III, open-label, and 14-week study (NCT02607280) enrolled 35 renal-impaired individuals with DPNP or PHN. Drug administration was based upon degree of renal impairment (moderate: 7.5 mg BID; severe: 7.5 mg QD). For the first 2 weeks, titration dose was applied, and for the subsequent 12 weeks, a fixed dose was applied [37]. The observed adverse effects primarily included nasopharyngitis (22.9%) and somnolence (11.4%) but only to a mild or moderate degree, implying the good tolerance performance of the drug. The secondary endpoint showed significantly decreased ADPS from baseline in patients with renal impairment, and LSM change was -1.9 (95% CI (-2.8, -1.0)). The mean standard deviation (SD) changes from baseline in ADSIS at week 14 were -1.4 (1.6) in patients with moderate symptom and -0.5 (0.7) in patients with severe symptom. In spite of decreased dose, mirogabalin markedly improved PGIC scores and diminished VAS and aggregate scores over the 14-week treatment.

#### 3.3.4. Fibromyalgia (FM)

In three double-blind, phase III studies, 3864 patients with FM were observed for 13 weeks in randomly allocated groups of placebo, pregabalin 150 mg BID, mirogabalin 15 mg QD, or mirogabalin 15 mg BID (NCT02146430, study A, 2318; NCT02187159, study B, 2280; NCT02187471, study C, 2526) [38]. No statistical significance was demonstrated in ADPS at week 13 for mirogabalin of either dose or the impact of mirogabalin in comparison with placebo on primary secondary endpoints, which included PGIC, FIQ, BPI-SF severity score, BPI-SF worst pain score, MFI-20, SF-36 physical component, and sleep disorder. The observed TEAEs in mirogabalin group were dizziness (15.2%), encephalalgia (13.5%), somnolence (9.6%), weight gain (8.7%), and nausea (8.0%).

Security of mirogabalin on a long-term basis for FM therapy was evaluated in a 52-week extension study (NCT02234583). Patients completed 13-week treatment and were given open-label mirogabalin 15 mg QD during the first 3 weeks of the trial period and 15 mg BID for titration period. The two groups retained the reduction in ADPS over the trial period, and because without statistical support, no conclusion can be reached concerning the long-term pain relief efficacy, notably when placebo is absent in the case. No unexpected adverse events were reported.

#### 3.3.5. Other Diseases

A double-blind phase III study of mirogabalin for the treatment of central NeP after spinal cord injury has achieved its primary endpoint (NCT03901352) [39]. With 274 subjects from Asia, the study concluded that ADPS changes 14 in the placebo group, indicating that mirogabalin outdid placebo and met the primary endpoints [39]. No additional safety concerns were observed.

A 14-week multicenter, randomized, double-blind, and placebo-controlled phase III study of mirogabalin for DPNP treatment in China was at the initial stage of enrollment (NCT04094662) [40]. A randomized, open-label, and interventional study investigated the efficacy and safety of mirogabalin in combination with conventional therapy for neuralgia after thoracic surgery (Japan Registry of Clinical Trials Identifer: jRCTs071200053) that is under way at present. Research data will see publication in the near future [41].

### 3.4. Case Reports

Two cases focused on rare adverse reaction, and 2 cases focused on the treatment of other diseases, including secondary trigeminal neuralgia, trigeminal trophic syndrome (TTS), and neuropathic itch-associated prurigo nodules, until December 2021 (Table 3).

A 77-year-old female patient diagnosed with lung cancer squamous cell carcinoma was reported to develop neutropenia at week 7 under a dosage regimen of 10 mg/day mirogabalin, which was suspected to be an aftermath of mirogabalin [42]. The symptom disappeared one week after the patient discontinued the drug. In effect, prior to this, mirogabalin's packaging description indicated a case of neutropenia; however, this case was not published in a case report. Drug-induced neutropenia occurred generally 19-60 days of pregabalin use, which might be caused by myeloid cell impairment depending on dosage [43]. In another case [44], a 48-year-old female with trigeminal neuralgia was prescribed mirogabalin 2.5 mg/d. Although she felt some relief 3 days later, treatment was obliged to be discontinued because of reported dizziness and drowsiness.

The third case was concerned with the off-label use of mirogabalin. TTS is a rare facial ulceration usually occurring after the damage of the trigeminal nerve or its central sensory connections. A female patient, 89, was not in the condition of taking antihistamines (loratadine 20 mg and fexofenadine 60 mg QD) on account of dizziness [45]. Mirogabalin (2.5 mg BID, weekly ascending to 7.5 mg BID) proved to be safe in this patient, and her pain mitigated from 5 to 4, and her itching significantly eased from 5 to 1 on numerical rating scales (NRS). In addition, increased doses of mirogabalin were considered a possible utility for TTS treatment. Similarly, in another case, a Japanese male patient, 73, with herniated cervical discs for 8 months and severe itch for 6 month, received treatment of mirogabalin (10 mg/d), and fundamental improvement in prurient was detected at week 2 (NRS 1/10) and flattening of prurigo nodules at week 4 [46].

On the whole, decriminalization neurasthenia is an adverse effect that physicians should bear in mind in drug administration. The conclusion might not be finalized considering the small quantity of adverse effect reports, either for off-label treatment of other diseases or for rare adverse effects.

### 3.5. Cost-Effectiveness Studies

Mirogabalin was added as a drug for P-NeP in 2019 and was marketed as a drug employed for PHN and DPNP in Taiwan in 2020 [7]. Pregabalin was approved for the treatment of NeP and refractory epilepsy in 2004 and 2005, respectively [47, 48]. The evaluation of the cost-effectiveness of mirogabalin as opposed to other gabapentinoids in treating PHN was of vital importance.

Recently, two studies focused on the assessment of the cost-effectiveness of mirogabalin versus zero treatment or 300 mg of pregabalin for PHN or DPNP in Taiwan. Contrastive analysis indicated that mirogabalin (30 mg) proved to be more cost-effective than placebo in the case of PHN and DPNP. On the basis of the deterministic analysis, mirogabalin could result in a gain of 0.041 and 0.02 quality-adjusted life year at an incremental cost of U$365 and US$323 in PHN and DPNP patients versus placebo (ICER: $8900 and $15860/QALY, respectively). Aside from that, 30 mg mirogabalin was cost-effective in comparison with 300 mg pregabalin (ICER: $6535/QALY) in PHN patients. In the two studies, the cost-effectiveness acceptability curve indicated mirogabalin 30 mg as a good treatment option for PHN and DPNP with an incremental cost-effectiveness ratio below the willingness-to-pay threshold (WTP $56000/QALY, 2.5 times GDP) in Taiwan.

## 4. Discussion

Mirogabalin was considered to reveal the analgesic effect by cutting down on calcium current via binding to the *α*2*δ* subunit of VGCCs [49]. It demonstrated analgesic effects in the intermittent cold stress model mice (female mice) [50] and in the unilateral intramuscular acidic saline injection model rats (male rats) [51, 52] in animal experiments and played an inhibitory role in N-type calcium channel currents in rat dorsal root ganglion neurons [49]. It has been demonstrated that it blocks neuronal excitation and sensory signals by reducing calcium entry into nerve endings and reduces calcium-mediated release of excitatory neurotransmitters in the dorsal horn [53, 54].

Mirogabalin was rapidly absorbed with *T*_max_ of 0.5-1.5 h after administration with single or multiple doses [23]. The *C*_max_ and AUC_inf_ of mirogabalin with a single oral dose of 3-30 mg increased in a dose-proportional manner, and *T*_1/2_ value had a range of 2.96-3.37 h. Since bioavailability was similar in the fed and fasted states, there was no food restriction, but it appeared to delay the uptake of mirogabalin in the fed condition. Almost 2/3 dose of mirogabalin was excreted in the urine, with a renal clearance of 10.4-12.4 L/h. Following the administration of ^14^C-mirogabalin, almost 98% of cumulative excretion rate was tested 168 h after an administration of a single oral dose of ^14^C-mirogabalin in healthy male adults [25].

Mirogabalin was primarily excreted as the parent drug by renal secretions, and AUC_last_ in patients with renal impairment significantly increased in association with aggravation of renal function and decreased CLcr [26]. *C*_max_ of mirogabalin grew by 2%, 48%, and 31%, respectively, in patients with moderate and severe renal impairment and ESRD, as opposed to the control group. Total CL/F of mirogabalin was reduced by 25%, 54%, and 76% in subjects with mild, moderate, and severe renal impairment, respectively, relative to normal controls. On the whole, mirogabalin dose adjustment led to a 50% to 75% decrease with moderate or severe renal damage and ESRD [26].

Mirogabalin is generally administrated in the elderly P-NeP patients with damaged renal functions, and the morbidity of DPNP and PHN has some links with advanced age [37]. These common clinical conditions have prompted researchers to focus on the safety and tolerability of mirogabalin in patients with DPNP or PHN-related renal injury and pain. Following these researches, the daily dose should be adjusted, referring to creatinine clearance levels listed in the table below (Table 4). Patients with DPNP or PHN were recommended to use 7.5 mg QD (severe impairment) or 7.5 mg BID (moderate impairment) of mirogabalin. For patients with mild or moderate hepatic impairment, it had no significant effect (>two-fold) on mirogabalin exposure, and there was no apparent need for dose adjustment for mild or moderate cases [27].

NeP is a chronic and irreducible condition, and more often than not, enduring multiple medications was essential for the patients. Actually, there are chances that increased central nervous system- (CNS-) related side effects occur for pregabalin with coadministered drugs [29]. There is indeed a need to cover the evaluation on the drug-drug interaction with mirogabalin. As a third member of the gabapentinoids, increased effects of body sway and digit symbol substitution test assays, as well as increased CNS-related adverse effects, were observed when lorazepam or ethanol was administered with mirogabalin. Major transporters involved in the secretion of mirogabalin are organic anion transporter (OAT) 1/3, organic cation transporter (OCT) 2, multidrug and toxin extrusion (MATE) 1, and MATE 2-K^31^. Mirogabalin is also metabolized by uridine diphosphate-glucuronosyl transferases (UGTs) [55]. Two clinical trials showed that with coadministration of mirogabalin with OAT1/3 inhibitor, OCT2 or MATE inhibitor, the PK of mirogabalin might be altered generally [29, 30]. AUC_0-last_ and *C*_max_ of mirogabalin were increased, and mean SD CLcr for mirogabalin was substantially slower after coadministration of probenecid or cimetidine versus mirogabalin alone. The increased exposure to mirogabalin in combination with probenecid or cimetidine was consistent with the increase observed in patients with mild renal impairment [26]. Since mild renal impairment does not entail dose adjustment of mirogabalin, mirogabalin adjustment is not recommended in the combined administration with probenecid or cimetidine. Yet, it should be noted that some caution should be exercised if mirogabalin is in combined use with the abovementioned two drugs along with lorazepam or alcohol.

Table 5 shows the summary of phase II and phase III clinical trials on mirogabalin. As is known to all, mirogabalin has been approved for P-NeP, PHN, and DPNP, subsequently, in Asian countries. In a key phase II trial (NCT01504412), the placebo-adjusted LSM difference in SF-MPQ sensory, VAS score, and ADSIS was statistically significant in the 15 mg BID group (-1.9, 95% CI (-3.6, -0.2); *P* = 0.0313, -7.4, 95% CI (-13.0, -1.8); *P* = 0.0093, -0.9, 95% CI (-1.3, -0.4); and *P* = 0.0002, respectively) [33]. The above data are supportive of more benefits of mirogabalin in DPNP patients, in consistency with the foregoing findings in the literature. In two other noteworthy phase III trials (NCT02318706, NCT02318719), the study population was composed of a cohort of 834 patients with DPNP and 765 patients with PHN, and the mirogabalin at 20-30 mg/day, 30 mg/day in particular, was found to alleviate pain in the participants remarkably in comparison with placebo [35, 56]. The findings further confirmed the therapeutic action of mirogabalin in patients with DPNP or PHN. Although several clinical trials in patients with FM and other P-Nep have not reached a clear conclusion as to the security and efficacy of mirogabalin [38, 57], an increasing number of clinical studies are dedicated to relevant investigation and validation.

Mirogabalin is well tolerated with manageable adverse reactions. In two phase III studies, the frequency of adverse reactions increased with the daily dose, with 42.4% in the 20 mg/day group and 62.4% in the 30 mg/day group on DPNP patients and with 35.3% in the 20 mg/day group and 44.5% in the 30 mg/day group on PHN patients. The most frequently detected adverse effects included somnolence, fatigue, dizziness, weight gain, edema, and nasopharyngitis. This conclusion was confirmed by two other phase III studies, which showed that adverse reactions occurred in 27.6% (59/214) of DPNP patients and 39.7% (94/237) of PHN patients. Hepatic function disorder (AST or ALT increased) may also be detected by a thorough examination. In the presence of abnormalities, appropriate measures should be taken, including drug discontinuation.

## 5. Conclusion

Neuropathic pain is a chronic condition that is detrimental to individuals' health both physically and mentally, and nevertheless, merely a small fraction of patients could realize pain alleviation. Mirogabalin, an orally administered gabapentinoid, has won approval from the Japanese authority for the treatment of peripheral neuropathic pain and DPNP. It is therefore clinically vital to make a precise evaluation as to its efficacy and other indicators in comparison with the other two gabapentinoids. This review confirms the favorable analgesic activity of mirogabalin by unique binding characteristics to *α*2*δ*-1 and *α*2*δ*-2 of VGCCs. The completed phase I-III clinical trials demonstrated that an oral 30 mg/day dose of mirogabalin showed good tolerability in patients without severe adverse effects. Observed adverse effects included somnolence, fatigue, and dizziness but with a much lower occurrence rate than the other two gabapentinoids. A combined regimen of mirogabalin with food or CNS inhibitors showed no obvious impact on PK and PD parameters but the PK effect with lorazepam and ethanol showed a modest increase.

To date, phase III studies of mirogabalin for fibromyalgia and other NeP have achieved consistent outcomes, and follow-up research should investigate the optimization of mirogabalin in more treatment options and provides a wider array of therapeutic regimens for NeP patients. More evidence should be collected as to the application of mirogabalin with a view to giving full play to the medication in clinical practice.

## Figures and Tables

**Table 1:** Chemical property of mirogabalin.

Common name	Molecular formula	CAS number	Melting point	Molecular weight
Mirogabalin besylate	C_18_H_25_NO_5_S	1138245-21-2	169°C	367.46

**Table 2:** Coadministration effect and drug-drug interaction with mirogabalin.

No.	Author	Year	Race	Mirogabalin	Concomitant medicine		PK parameter of mirogabalin	Safety assessment	Conclusion
				Dose	Drug	Dose			
1	James Dow et al.	2018	Unknown	15 mg	Metformin	850 mg	PK parameters were similar when administered alone or in combination	Dyspepsia, encephalalgia, increased hepatic enzymes (AST and ALT were 2.7 and 2.5 × ULN, respectively).	Well tolerated with no evidence of a drug-drug interaction.

2	Mendel Jansen et al.	2018	Unknown	20 mg	Lorazepam	2 mg	PK parameters were similar when administered alone or in combination	Increased effects in body sway and DSST assays; increased occurrence of somnolence.	Potentially increased central nervous system-related AEs when lorazepam or ethanol was coadministered with mirogabalin.
					Zolpidem	10 mg	*C*_max_ decreased by 11%, (90% CI), 0.89 (0.82, 0.96)	Increased occurrence of somnolence.	
					Tramadol	100 mg	*C*_max_ decreased by 28%, (90% CI), 0.72 (0.67, 0.76)	Increased incidence of nausea.	
					Ethanol	240 mL men/200 mL women	*C*_max_ increased by 20%, (90% CI), 1.20 (1.12, 1.28)	Increased the PD effects in body sway and DSST assays; increased incidence of encephalalgia.	

3	Masaya Tachibana et al.	2018	White, Black, African American	15 mg	Probenecid	500 mg Q6H	AUC_0-last_ and *C*_max_ increased by 76% and 29%	No clinically significant changes in vital signs or ECGs.	No necessary to dose adjustment with administration of probenecid and cimetidine, since the effect was not significant (>2-fold).
					Cimetidine	400 mg Q6H	AUC_0-last_ and *C*_max_ increased by 44% and 17%		

ULN: upper limit of normal; Q6H: once every 6 h; DSST: digit symbol substitution test; AST: aspartate aminotransferase; ALT: alanine transaminase; ECGs: 12-lead electrocardiograms.

**Table 3:** Cases reported focusing on mirogabalin.

No.	Subject	Disease	Drug dose	Drug treatment	Treatment-related results
1	77 years (woman)	Lung carcinoma	10 mg/day	6 weeks	Mirogabalin-induced neutropenia
2	48 years (female)	Trigeminal neuralgia	2.5 mg/d	3 days	Mirogabalin-induced dizziness and drowsiness
3	89 years (female)	Trigeminal trophic syndrome	2.5 to 7.5 mg BID	Unknown	NRS from 5/10 to 1/10
4	73 years (man)	Neuropathic itch	10 mg/d	2 weeks	NRS from 9/10 to 1/10

**Table 4:** Dose of mirogabalin following renal impairment (creatinine clearance (CL_cr_) : L/min).

Mirogabalin	Mild (90 > CL_cr_ ≥ 60)	Moderate (60 > CL_cr_ ≥ 30)	Severe (including ESRD) (30 > CL_cr_)
Daily dose	10-30 mg	5-15 mg	2.5-7.5 mg
Initial dose	5 mg BID	2.5 mg BID	2.5 mg QD
Fixed dose	15 mg BID	7.5 mg BID	7.5 mg QD

**Table 5:** Summary of phase II and phase III clinical trials published about mirogabalin.

No.	Author/year	Disease	Study	Drug doses	Enroll	Race	Study endpoint	Serious AEs (SAEs) in the mirogabalin groups
1	Aaron Vinik/2014	DPNP	Phase II (NCT01496365)	Mirogabalin 5, 10, 15 mg, QD, 10, 15 mg, BID; placebo; pregabalin 150 mg BID for week 5	452	White; Black/African American; others	Mirogabalin groups were well tolerated and had statistically significant reductions in ADPS versus placebo.	One case: ALT level > 3X ULN, AST > 3X ULN, and total bilirubin level > 2X ULN in 15 mg/day group.
2	Domenico Merante/2017	DPNP	Phase II (NCT01496365)	Mirogabalin 5, 10, 15, 20, 30 mg; placebo; pregabalin 300 mg/day for week 5	452	White; Black/African American; others	Mirogabalin groups were significant reductions in ADSIS, compared with placebo (*P* < 0.05). The percentage of subjects for the PGIC was greater in the each mirogabalin groups than that in the placebo group (31.1%) (*P* < 0.05).	One case: ALT level > 3 × ULN, AST > 3 × ULN, and total bilirubin level > 2 × ULN in 15 mg/day group.
3	Masayuki Baba/2020	DPNP	Phase II (NCT01504412)	Mirogabalin 5, 10, 15 mg, BID; placebo; pregabalin 150 mg BID for week 7	450	Asian	Mirogabalin groups were a greater improvement in ADPS, compared with placebo, although having no statistically significant. Mirogabalin 15 mg BID significantly improved the SF-MPQ sensory and visual analog scale scores and ADSIS, versus placebo (*P* < 0.05).	No mentioned.
4	Masayuki Baba/2020	DPNP	Phase III (NCT02318706)	Placebo, mirogabalin 15, 20 or 30 mg/day for up to 14 weeks, with a 1- to 2-week titration	834	Asian	LSM change from baseline in ADPS for mirogabalin 30 mg/day was statistical significance reduction compared with placebo (*P* = 0.0027). Reductions in VAS and in ADSIS were statistically significant for mirogabalin 30 mg/day versus placebo (*P* = 0.0018, *P* = 0.0001), respectively. Significantly, more patients treated with mirogabalin 30 mg/day reported a PGIC score, compared with placebo. (*P* < 0.05).	Two cases: severe dizziness or edema in 15 mg/day group. One case: alanine transferase and hepatic enzyme increased in 15 mg/day group.
5	Masayuki Baba/2020	DPNP	Extension study followed phase III (NCT02318706)	An initial dose of 5 mg BID for initial 2 weeks, 10 mg BID for the second 2 weeks, and increased to dosage of 10 or 15 mg BID for the reminding weeks.	214	Asian	All SF-MPQ scales, including sensory score, affective score, total score, VAS, and present pain intensity, decreased from baseline to week 52.	One case: aspartate aminotransferase increased.
6	Jitsu Kato/2019	PHN	Phase III (NCT02318719)	Mirogabalin 15 mg QD, 10 mg BID, or 15 mg BID; placebo for 14 weeks	765	Asian	Mirogabalin groups were a statistically significant difference in mean change in ADPS from baseline, compared with placebo (*P* < 0.05). LSM change from baseline to week 14 in VAS of the SF-MPQ and the ADSIS was significantly greater in all mirogabalin groups compared with placebo (*P* < 0.05). Significantly, more patients treated with each mirogabalin reported a PGIC score, compared with placebo (*P* < 0.05).	Pneumonia, rib fracture, and femur fracture in 15 mg/day group; erectile dysfunction, fracture, and upper limb fracture in 20 mg/day group; increased blood creatine phosphokinase, memory impairment, altered state of consciousness, cerumen impaction, and electrocardiogram change in 30 mg/day group.
7	Jitsu Kato/2020	PHN	Extension study followed phase III (NCT02318719)	An initial dose of 5 mg BID for initial 2 weeks, 10 mg BID for the second 2 weeks, and increased to a flexible maintenance dosage of 10 or 15 mg BID for the reminding weeks.	239	Asian	All SF-MPQ scales, including sensory score, affective score, total score, VAS, and present pain intensity, decreased from baseline to week 52.	A case: severe AE of dizziness. Two cases: 12-lead electrocardiogram abnormalities (atrial flutter and acute myocardial infarction).
8	Masayuki Baba/2020	Renal impairment with DPNP or PHN	Phase III NCT02607280	7.5 mg BID for moderate impairment and 7.5 mg QD for severe impairment	35	Japanese	Significantly decreased ADPS from baseline in patients with renal impairment.	No unexpected adverse.
9	Lesley M. Arnold/2019	FM	Phase III (NCT02146430); (NCT02187159); (NCT02187471) Extension study (NCT02234583)	Mirogabalin 15 mg QD; mirogabalin 15 mg BID; placebo; pregabalin 150 mg BID	3864	North America, Asia Pacific, Eastern Europe, Western Europe, and Latin America	No statistical significance for the change in ADPS at week 13 at mirogabalin groups, as well as the effect on key secondary endpoints. Conclusions cannot be drawn regarding the long term effect of mirogabalin on pain.	No unexpected adverse.

## Data Availability

The data used to support the findings of this study are included within the article.
